# 

**Published:** 1906-06-15

**Authors:** 


### CONTENTS.

Event and Comment - - - - - - - - - - - 271
Hemophilic and Secondary Hemorrhages, By George Zederbaum, D.D.S. 275
Filling Molars that Require Devitalization, By P. F. Hines, D.D.S. 281
Value of the Dental Society, - - - By R. W. Morse, D.D.S. 284
When to Devitalize, - - - - By J. E. Stoffer, D.D.S. 286
What Does the Dental Profession Most Need?
By Truman W. Brophy, M.D., D.D.S., L.L.D. 289
With Our Editors - - - - - - - - - - 294
Indents - - - - - - - - - - - - 303
Announcements - - - - - - - - - - 317
Obituary, - - - - - - - - - - - - - 322

